# Evaluating How Safety-Net Hospitals Are Identified: Systematic Review and Recommendations

**DOI:** 10.1089/heq.2021.0076

**Published:** 2022-04-14

**Authors:** W. Ryan Powell, Kellia J. Hansmann, Andrew Carlson, Amy J.H. Kind

**Affiliations:** ^1^ Center for Health Disparities Research, University of Wisconsin School of Medicine and Public Health, Madison, Wisconsin, USA.; ^2^ Department of Family Medicine and Community Health, University of Wisconsin School of Medicine and Public Health, Madison, Wisconsin, USA.; ^3^ University of Wisconsin School of Medicine and Public Health, Madison, Wisconsin, USA.; ^4^ Geriatrics Division, Department of Medicine, University of Wisconsin School of Medicine and Public Health, Madison, Wisconsin, USA.

**Keywords:** safety-net hospital, socioeconomic disadvantage, social determinants of health, health equity, health policy, hospital performance

## Abstract

**Objective:**

To systematically review how safety-net hospitals' status is identified and defined, discuss current definitions' limitations, and provide recommendations for a new classification and evaluation framework.

**Data Sources:**

Safety-net hospital-related studies in the MEDLINE database published before May 16, 2019.

**Study Design:**

Systematic review of the literature that adheres to Preferred Reporting Items for Systematic Reviews and Meta-Analyses (PRISMA) guidelines.

**Data Collection/Extraction Methods:**

We followed standard selection protocol, whereby studies went through an abstract review followed by a full-text screening for eligibility. For each included study, we extracted information about the identification method itself, including the operational definition, the dimension(s) of disadvantage reflected, study objective, and how safety-net status was evaluated.

**Principal Findings:**

Our review identified 132 studies investigating safety-net hospitals. Analysis of identification methodologies revealed substantial heterogeneity in the ways disadvantage is defined, measured, and summarized at the hospital level, despite a 4.5-fold increase in studies investigating safety-net hospitals for the past decade. Definitions often exclusively used low-income proxies captured within existing health system data, rarely incorporated external social risk factor measures, and were commonly separated into distinct safety-net status categories when analyzed.

**Conclusions:**

Consistency in research and improvement in policy both require a standard definition for identifying safety-net hospitals. Yet no standardized definition of safety-net hospitals is endorsed and existing definitions have key limitations. Moving forward, approaches rooted in health equity theory can provide a more holistic framework for evaluating disadvantage at the hospital level. Furthermore, advancements in precision public health technologies make it easier to incorporate detailed neighborhood-level social determinants of health metrics into multidimensional definitions. Other countries, including the United Kingdom and New Zealand, have used similar methods of identifying social need to determine more accurate assessments of hospital performance and the development of policies and targeted programs for improving outcomes.

## Introduction

Safety-net hospitals serve a higher proportion of patients from disadvantaged populations and are increasingly the focus of research investigating access to care, health outcomes, health care quality, and hospital financial performance. Yet a standard definition for identifying and evaluating these hospitals does not exist.^1^ How these hospitals are identified and categorized influences hospital measurement and performance which has important policy consequences impacting their financial viability and stability.^2^

For example, nearly all types of safety-net providers from various clinical settings (e.g., hospital, physician group, dialysis, Accountable Care Organizations, and Medicare Advantage contracts) were more likely to be penalized or not receive bonuses due to worse performance on quality measures within all nine value-based purchasing programs according to a 2016 report to Congress by the Office of the Assistant Secretary for Planning and Evaluation within the Department of Health and Human Services.^3^

The programs included the Hospital Readmissions Reduction Program, the Hospital-Acquired Conditions Reduction Program, the Hospital Value-Based Purchasing Program, the Medicare Advantage Quality Star Rating Program, the Medicare Shared Savings Program, the Physician Value-Based Payment Modifier Program, the End-Stage Renal Disease Quality Incentive Program, Skilled Nursing Facility Value-Based Purchasing Program, and Home Health Value-Based Purchasing Program.

Yet, current definitions of safety-net hospitals used in research and policy are not only varied, they may also be incomplete. Health services researchers and policy makers are increasingly debating the extent to which patient and community factors outside the hospital influence hospital performance measures for value-based purchasing programs.^3–9^ Health disparities theory is central to the argument for including measures of social disadvantage in health services research and policy.

For instance, the National Institute of Aging's Health Disparities Research Framework emphasizes the importance of considering the environmental, sociocultural, behavioral, and biological factors that shape health and lead to health disparities.^10^ Ignoring social conditions that are fundamental causes of disease—such as factors related to financial wealth and stability, education, the social and community context, and the neighborhood and built environment—can perpetuate health inequities and lead to missed opportunities to promote health benefits across populations.^11–14^

In particular, the debate of whether social risk factors should be considered when determining hospital readmission penalties under Medicare's Hospital Readmissions Reduction Program has been widely discussed.^8,9,15–22^ Risk of financial penalties is a particular concern for hospitals serving large proportions of disadvantaged populations, since such populations have disproportionately higher readmission rates and face specific non-hospital challenges after discharge, including socioeconomic constraints and lack of social support.^4,16,23–27^

Adjusting for the contribution of social risk factors to 30-day hospital readmissions is projected to potentially reduce Hospital Readmission Reduction Program penalties for a majority of safety-net hospitals and would result in a total decrease in penalties to safety-net hospitals of more than $17 million.^28^ The combination of undue penalties, lower reimbursement rates, and often thin financial margins may result in these hospitals having fewer resources to invest in the quality improvement efforts needed to avoid future penalties. This amplifies a troubling pattern of “poor performance and little chance for improvement.”^9,29^

Recent changes to Centers for Medicare & Medicaid Services (CMS) policies potentially lighten penalties to those hospitals serving higher proportions of patients with Medicaid coverage. But Medicaid coverage alone is likely not an adequate surrogate for disadvantage given its legal variations and state-by-state differences in eligibility. The use of such a metric may place hospitals within certain states at an arbitrary measurement disadvantage, resulting in penalties for otherwise equal performance. The wide array of potential measures underscores the need for a standard definition.^30–32^

The aim of this systematic review was to characterize the array of operational definitions used to identify safety-net hospitals for the purpose of original research. We evaluated methodologies used to summarize disadvantage at the hospital level for their strengths and limitations from the perspective that social conditions are a fundamental cause of disease and must be addressed to effectively reduce health disparities and improve health outcomes.^33^ Understanding the breadth of potential definitions of safety-net hospitals is essential to creating a more uniform and precise framework for identifying safety-net hospitals that incorporates findings from previous studies as well as current conceptual models of health equity.

### New contribution

Since the enactment of the Affordable Care Act in 2010, and its associated increase in CMS value-based payment programs, there has been increased research and policy interest in safety-net hospitals that care for higher proportions of patients from disadvantaged populations. Yet no systematic review including studies since 2010 has been conducted to understand how these hospitals are identified or how disadvantage is summarized at the hospital level. In this review, we evaluate the existing approaches and discuss opportunities for increased precision and consistency of an identification framework.

In particular, we provide evidence for how a new framework could provide a better reflection of the real-world disadvantage faced by patients. A standard definition for identifying safety-net hospitals will inform research and policy in new ways, which may in turn facilitate better-informed health system decisions and refinements around hospital performance measures. Key insights from the results of our review and from established health equity theory underscore the need for a multidimensional definition of disadvantage that includes individual and community-level social risk factors.

## Methods

### Study identification and selection

Studies were eligible for inclusion in our review if they were original research; described an operational definition for identifying safety-net hospitals; compared more than one safety-net hospital; and were published in English.

We searched the MEDLINE database (through PubMed) from inception through May 16, 2019 using the search terms: (“safety-net hospitals”[All Fields] OR “safety-net hospital”[All Fields]) AND English[Language]. Reference lists were also hand searched for other relevant studies. A total of 740 articles from MEDLINE and hand searches of reference lists were reviewed for inclusion into the systematic review. PRISMA reporting guidelines were followed. All articles were randomly assigned to six independent reviewers and reviewed for eligibility.

If an abstract met the inclusion criteria described earlier—specifically if it involved original research comparing two or more safety-net hospitals—then a full-text review followed. Subsequently, if the full-text article described an operational methodology to identify and define safety-net hospitals (or referenced an existing method), then the article was included in the systematic review. Based on abstract reviews of the original 740 studies, 273 were then full-text reviewed for eligibility. Of those, a total of 132 met the requirements for inclusion into the systematic review (see Supplementary Fig. S1 for a flow diagram of the study selection process).

### Data extraction, review, and reporting from full-text articles

All articles meeting inclusion criteria were fully reviewed by two separate reviewers, whereby they first reviewed the same 10 randomly selected articles for training purposes to confirm concordance, then independently extracted the remaining articles separately. Using an established rubric, information was then extracted about the method used to define safety-net hospitals for each study.

This included details about the topic area of the study's objective (evaluating access to care, health outcomes, health care quality metrics, health system financial performance, etc.); study methods including the data source, the operational definition for identifying safety-net hospitals, each of the dimensions of disadvantage used; and details of the study's analysis plan including the unit of analysis and how the operational definition was categorized and used in the study's analyses.

Authors of individual articles were not contacted to obtain further information. Each extraction element was compared using Cohen's Kappa to assess inter-rater reliability. Kappa statistics had almost perfect agreement for most definition dimensions, the data source, and for items indicating how the definition was used in analyses. There was moderate agreement for the method's unit of analysis (Kappa=0.56). Inter-rater reliability for the health care topic ranged from fair to almost perfect (range: 0.37–0.82). Any disagreements were resolved using a second joint review.

## Results

### Search results and study characteristics

Research on safety-net hospitals increased precipitously (4.5-fold) in the past decade: Our review yielded 23 studies published between 1997 and 2010; but after 2010 through May 2019, another 109 studies comparing safety-net hospitals were published (Supplementary Fig. S2). The Supplementary Data to this review includes a table summarizing each study's methodology and includes a description of the safety-net hospital definition, its data source, and how it was used in the analysis.

Table 1 describes the topic areas of the studies' objectives. Of the studies included in this review, 65% focused on questions related to quality of care (17% structure, 13% process, and 46% outcome domains), 50% relevant to access to care, 35% evaluating hospital financial performance, and 8% on the cost of care.

**Table 1.** Summary of Study Characteristics (***N***=132)

Study topic type	Overall ***n*** (%)	Unidimensional definition ***n*** (%)	Multidimensional definition ***n*** (%)
Access to care	66 (50.0)	11 (37.9)	55 (53.4)
Quality of care	86 (65.2)	22 (75.9)	64 (62.1)
Structure	23 (17.4)	5 (17.2)	18 (17.5)
Process	17 (12.9)	5 (17.2)	12 (11.7)
Outcome	61 (46.2)	15 (51.7)	46 (44.7)
Hospital financial performance	46 (34.8)	6 (20.7)	40 (38.8)
Costs of care	11 (8.3)	1 (3.4)	10 (9.7)

### Safety-net definitions and disadvantage dimensions

We first assessed the operational definition of safety-net hospitals used by each study by whether it incorporated one or more dimensions of disadvantage (Table 2). The majority of studies used a multidimensional definition to identify safety-net hospitals: 86 studies (65.2%) described a definition with two dimensions of disadvantage and 17 studies (12.9%) used three or more dimensions. The remaining 29 studies (22.0%) involved a definition with only one dimension of disadvantage.

**Table 2.** Dimensions of Disadvantage Captured in Existing Safety-Net Definitions (***N***=132)

Dimension characteristics	Overall ***n*** (%)	Unidimensional definition ***n*** (%)	Multidimensional definition ***n*** (%)
Number of dimensions used			
1	29 (22.0)		
2	86 (65.2)		
3 or more	17 (12.9)		
Disadvantage dimensions			
Payer mix	106 (80.3)	16 (55.2)	90 (87.4)
Medicaid	100 (75.8)	10 (34.5)	90 (87.4)
Uninsured	50 (37.9)	6 (20.7)	44 (42.7)
Private insurance	1 (0.8)	0 (0)	1 (1.0)
Uncompensated care	15 (11.4)	0 (0)	15 (14.6)
Charity care	22 (16.7)	0 (0)	22 (21.4)
DSH index	27 (20.5)	0 (0)	27 (26.2)
SSI	28 (21.2)	1 (3.4)	27 (26.2)
Association membership	10 (7.6)	6 (20.7)	4 (3.9)
Hospital characteristic(s)	21 (15.9)	5 (17.2)	16 (15.5)
Minority	4 (3.0)	0 (0)	4 (3.9)
Income	4 (3.0)	0 (0)	4 (3.9)
Education	2 (1.5)	0 (0)	2 (1.9)
Poverty	3 (2.3)	0 (0)	3 (2.9)
Homelessness	1 (0.8)	0 (0)	1 (1.0)
Unemployment	1 (0.8)	0 (0)	1 (1.0)
Owner-occupied homes	1 (0.8)	0 (0)	1 (1.0)
Homes with crowding	1 (0.8)	0 (0)	1 (1.0)
Self-identify	1 (0.8)	1 (3.4)	0 (0)
Other public low-income program	1 (0.8)	0 (0)	1 (1.0)

DSH, Disproportionate Share Hospital; SSI, Supplemental Security Income.

In total, our systematic review uncovered 18 factors (Table 2)—including hospital payer mix, hospital compensation, and specific dimensions of individual or community disadvantage—that were combined into 27 unique combinations to define and evaluate safety-net hospitals. Across all definitions, identifying safety-net hospitals by hospital payer mix (Medicaid, uninsured, or private insurance) was the most common (80%).

Specifically, Medicaid coverage was the most frequent dimension included in safety-net definition, employed in 76% of all definitions. There was heterogeneity in the way this dimension was measured (e.g., as percentage of discharges, costs, and patients), and in the data source used. Common sources were the American Hospital Association (AHA) annual survey, the Healthcare Cost and Utilization Project's (HCUP) National Inpatient Sample, and CMS's Hospital Inpatient Prospective Payment System Impact File.

Other dimensions were common. The extent to which hospitals serve the uninsured (38% of studies) was used to measure the extent to which a hospital serves disadvantaged populations. Some studies identified uncompensated care (11%) and one of its components, charity care (17%), as part of their definition—most frequently using hospital financial measures within the American Community Health survey.

In addition, several studies identified characteristics of the hospital such as public ownership and teaching status (16%) in their definition, and another 8% of studies identified being a member of an association (e.g., America's Essential Hospitals) as part of their definition. Remaining measures were used in <5% of the studies' definitions and included measures found in the American Community Health survey items at a five-digit zip-code level (racial and ethnic minority, income, poverty, education, homelessness, unemployment, owner occupied homes, homes w/crowding) or from a survey of hospital leaders that self-identified as safety-net hospitals.

The most common definition incorporated two dimensions of payer mix: Medicaid and Uninsured (29%). Another common multidimensional definition included the Disproportionate Share Hospital (DSH) index, which is a function of a hospital's total inpatient days from patients on Supplemental Security Income (SSI) with Medicare and the total inpatient days from non-Medicare patients on Medicaid. This definition was used exclusively in 18% of studies, often obtained through CMS Impact File Hospital Inpatient Prospective Payment system data or other CMS cost reports. Contextual neighborhood measures of income, education, housing quality, and employment were rarely considered.

### Safety-net comparison strategies

Across all approaches, there was substantial heterogeneity in how researchers categorized and compared the extent to which a hospital served disadvantaged populations (Table 3). Definitions relied on categorical groupings to classify safety-net status in various ways: 64% used percentile cutoffs (e.g., based on the distribution of DSH payments), 23% used a yes/no distinction (e.g., on/not on association member list), and 15% used a proportion of the total (typically for payer mix).

**Table 3.** Safety-Net Definition Methodological Strategies (***N***=132)

Methodological characteristics	Overall ***n*** (%)	Unidimensional definition ***n*** (%)	Multidimensional definition ***n*** (%)
Safety-net status classification approach			
Percentile ranking	84 (63.6)	9 (31.0)	75 (72.8)
Yes/no indicator	30 (22.7)	12 (41.4)	18 (17.5)
Proportion	20 (15.2)	2 (6.9)	18 (17.5)
Standard deviation	20 (15.2)	5 (17.2)	15 (14.6)
Ratio	7 (5.3)	1 (3.4)	6 (5.8)
Continuous/sliding scale	0 (0)	0 (0)	0 (0)
Analytic comparison approach			
Safety-net vs. non-safety-net	96 (72.7)	29 (100.0)	67 (65.0)
3 ordinal categories	23 (17.4)	0 (0)	23 (22.3)
4 ordinal categories	12 (9.1)	0 (0)	12 (11.7)
10 ordinal categories	1 (0.8)	0 (0)	1 (1.0)

Furthermore, all analyses assumed a threshold effect on study outcomes to categorize differences between hospitals on their disadvantage metric, with most analysis evaluating hospital outcomes based on a simple two category comparison—safety versus non-safety-net (73%). The remaining 27% of studies used >2 ordinal categorizations (e.g., low, medium, and high safety-net burden) to make comparisons. No approach treated disadvantage as a continuous metric or a “sliding-scale” measure of safety-net status.

## Discussion

Results of this systematic review reveal substantial variation between safety-net hospital definitions used to summarize the extent to which hospitals serve disadvantaged populations. Most studies frequently evaluated disadvantage by payer mix, primarily the extent to which a hospital serves those with Medicaid coverage or the uninsured. Other common approaches involved using uncompensated care and hospital characteristics. These approaches capture some aspects of disadvantaged populations, but findings raise several methodological concerns.

### Studies have continued to use imprecise unidimensional definitions

The reliance on any one measure of service to disadvantaged populations is problematic (for an in-depth discussion see McHugh et al.^2^) and not in alignment with modern health equity theory. Prior research suggests such unidimensional approaches should not be considered, yet were implemented in 22% of all research study definitions and are frequently used in health policy.^34,35^ Medicaid coverage was the most commonly used dimension of disadvantage, but as an indicator it has serious limitations since eligibility qualifications for Medicaid vary by state.

Tying definitions solely based on Medicaid coverage to national policy decisions will introduce measurement bias and increase penalty risk for hospitals purely due to their state of operation. For example, hospitals serving disadvantaged populations are at greater risk of financial penalties under the CMS Hospital Readmissions Reduction Program. Even while recent changes have been made to the program to adjust for the extent to which hospitals serve Medicaid beneficiaries, state-by-state differences in Medicaid eligibility requirements will lead to hospitals experiencing disproportionate impact.^36^

Hospitals serving a high proportion of disadvantaged populations in a state where Medicaid was not expanded may be more heavily penalized than those from states that expanded Medicaid. In addition, uncompensated care is tied to Medicaid^37^ and Medicaid expansion: states that expanded Medicaid and Medicare services also saw reduction in uncompensated care.^38–40^ This would impact safety-net hospital research and policy using an uncompensated care only definition.

As a unidimensional proxy for socioeconomic disadvantage and low income, Medicaid is also incomplete since not all who are disadvantaged receive SSI or are covered under Medicaid. Common measures used in studies such as the Disproportionate Share Hospital index (used in 20% of studies) may be capturing a specific and potentially skewed subgroup of low-income individuals. In Medicare, nearly 10% of the non-Medicaid population over a 10-year period will spend down their assets to qualify for Medicaid, for example, because of high costs for long-term services and support—this group represents an estimated two-thirds (64.2%) of those joining the Medicaid population during that period.^41^ This measure does not factor in older patients who are also less affluent but covered by Medicare.^42^ Similarly, reliance on organizational characteristics (e.g., public ownership and teaching status) revealed wide variation among disadvantaged measures related to uncompensated care and disproportion share hospital index, suggesting hospital characteristics alone are not an accurate indicator of safety-net status.^35,43^

### Multidimensional definitions are preferable but may still lack precision

Although most studies took a multidimensional approach by including two dimensions of disadvantage (most often Medicaid and the uninsured, or Medicaid and SSI), they rarely considered measures of disadvantage captured outside hospital walls that affect health. Factors such as the physical environment where patients live, public safety, social environment, social support, and other geographical and political considerations are largely out of a hospital's control without significant investment in resources to achieve equity.

Failure to capture the nuances of disadvantage beyond the health care system itself ignores important dimensions of disadvantage that drive disparities in health outcomes. Incorporating such factors would strengthen existing multidimensional approaches. Weaknesses of existing definitions and lack of standardization can obscure the true impact on key hospital performance measures.^15,22,26,28,44^ Inconsistent and inconclusive evidence might be due to a combination of measurement error and incomplete constructs, even when they are multidimensional.

### Strengthening future research and policy: Call for incorporating a health equity framework

A definition and evaluation framework guided by health equity theory and inclusive of a broader list of determinants of health is essential. Much needed attention is already underway to provide guidance on potentially feasible adjustments of social risk factors related to value-based payments; all consistent with a health equity framework.^6^ Some of the factors posited include socioeconomic position (income, education, dual Medicare/Medicaid eligibility, and wealth), race, ethnicity and cultural context (nativity and acculturation), gender, social relationships, residential, and community context.^6^ Such works highlight the incompleteness of current research and policy practices.

One simple strategy to begin to incorporate more nuance in definitions of hospital disadvantage is the neighborhood context where patients live (using geographically discrete aggregated data such as census block group or nine-digit zip code). Neighborhood disadvantage is a key driver of health and health care inequities,^11,25,33,45–47^ yet the neighborhood context of patients receiving care at the hospital was rarely considered in the studies included in this review. Social determinants of health are the “conditions in the environments in which people are born, live, learn, work, play, worship, and age that affect a wide range of health, functioning, and quality-of-life outcomes and risks.”^48^

Much of the current conversation has been focused identifying various low-income populations, but social determinants of health also incorporate additional dimensions of individual-level socioeconomic status (education and employment) as well as other contextual-level social risk factors such as safe housing, public safety, local food markets, access to health care services, and social support.^48^ Neighborhood context is an aggregate dimension of disadvantage that can strengthen the understanding of the disadvantaged populations hospitals may serve.

Prior study argues there are separate but overlapping disadvantaged subpopulations that can be captured with multidimensional approaches that include measures of social determinants of health.^35,43^

Zwanziger and Khan's^35^ concurrent evaluation of several definitions using Medicaid, uncompensated care and hospital service area socioeconomic disadvantage suggests these dimensions overlap but only slightly, as evidenced by a moderate correlation between hospital service area socioeconomic disadvantage and Medicaid, and small correlations between uncompensated care and hospital service area socioeconomic disadvantage and also between hospital service area socioeconomic disadvantage and Medicaid. This suggests neighborhood context confers risks beyond individual-level markers of disadvantage and has implications for how policymakers approach risk adjustment.

Until recently precision public health technologies that would allow such geo-linkages of social determinants of health were not widely available given technical limitations or access to neighborhood-level data. With the introduction of new precision public health resources (e.g., the NIH-funded Area Deprivation Index contained within the public Neighborhood Atlas^49^), researchers are now able to capture socioeconomic disadvantage at very granular neighborhood geographic levels. For example, census block group-level measures can be employed instead of previously used five-digit zip code-level measures (which can introduce considerable measurement error) in evaluating health associations.^50–52^

Moving forward, a standard definition should consider a larger set of social determinants of health based on health disparities theory and move beyond the health system silo. Relative to Medicaid payer mix, an advantage of incorporating precision public health metrics such as neighborhood disadvantage is that they are relatively stable measures over time when aggregated to the hospital level.^35,53^ Recent findings also support the significance of these measures to value-based payment programs such as penalties in the Hospital Readmission Reduction Program^28^ or the Merit-based Incentive Payment System scores in the ambulatory care setting.^54^

In summary, more precision and incorporation of health equity theory in characterizing safety-net hospitals is needed. From a hospital perspective, this could drive better risk adjustment to value-based purchasing program metrics (providing more accurate reflections of hospital performance), which may reduce penalties for those serving a disproportionate amount of disadvantaged populations. This in turn could result in hospitals having greater capacity to improve the health of disadvantaged populations through greater investment in clinical quality improvement programs and community health partnerships.

All of this is proposed with the goal of breaking the unintended consequence of policy penalties that sustain a pattern of poor performance coupled with little chance for improvement.^16^ Although long-term solutions that address social determinants of health are difficult yet fundamental,^33,55^ there is an opportunity to make policy improvements in the short term that are rooted in better research and more precise evaluations of hospital performance.

A framework for a standard definition should include the following elements:

(a) Employment of measures that are independently captured outside of health systems—that is, measures that do not solely rely on Medicaid coverage, uncompensated care (which varies by state and are inter-related), or hospital characteristics.
(b) A more holistic definition of disadvantage anchored to health equity theory using a multidimensional approach that includes precision public health measures and contextual-level as well as individual-level patient factors summarized to the hospital level. An emphasis on collecting and incorporating social determinants of health data into clinical decision-making, research, and policy has been discussed previously.^56–58^ Efforts to harmonize data collection during clinical assessments involve simple screenings that enable follow-up evaluations and link to appropriate support services.^56^ Detailed social-risk measures are increasingly available at the neighborhood level (census block group) for performance and policy evaluations. For example, population-based assessments of neighborhood-level disadvantage, which reflects the life challenges that make attaining health more difficult, can be feasibly incorporated into safety-net hospital definitions to improve measurement precision.^57^

## Supplementary Material

Supplemental data

Supplemental data

Supplemental data

## Abbreviations Used

CMS: Centers for Medicare & Medicaid Services
DSH: Disproportionate Share Hospital
HCUP: Healthcare Cost and Utilization Project
SSI: Supplemental Security Income
