# Supplementary material for: Evaluating How Safety-Net Hospitals Are Identified: Systematic Review and Recommendations
Source: Health Equity. 2022 Apr 14;6(1):298–306. doi: 10.1089/heq.2021.0076 (PMC9081065; doi:10.1089/heq.2021.0076)
Supplement: Supplemental data [file Suppl_FigureS1.docx]

Figure S1. Flow chart of study selection process for systematic review
