# Supplementary figures and images for: Evaluating How Safety-Net Hospitals Are Identified: Systematic Review and Recommendations
Source: Health Equity. 2022 Apr 14;6(1):298–306. doi: 10.1089/heq.2021.0076 (PMC9081065; doi:10.1089/heq.2021.0076)

Figure S2. Frequency distribution of safety net related studies over time through May 16, 2019


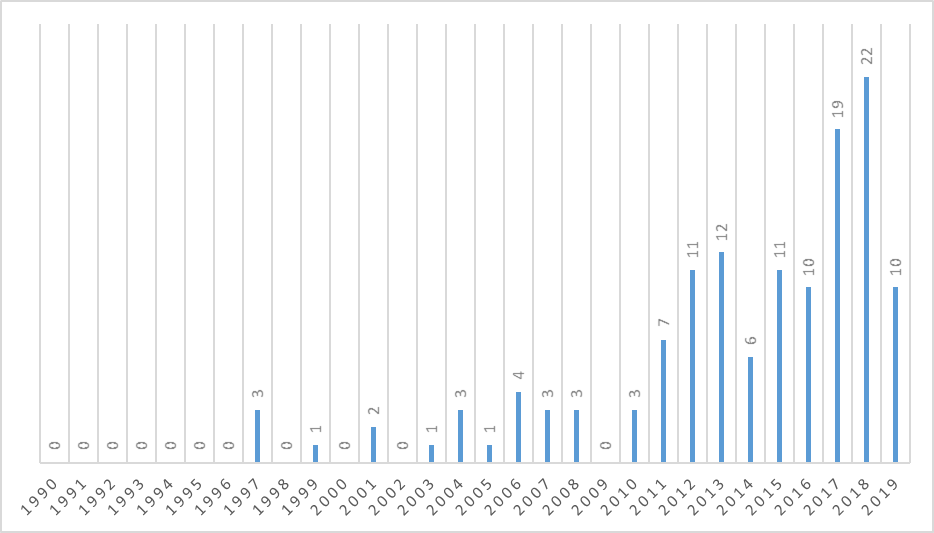

Supplement: Supplemental data [file Suppl_FigureS2.docx]
