# Supplementary material for: Evaluating How Safety-Net Hospitals Are Identified: Systematic Review and Recommendations
Source: Health Equity. 2022 Apr 14;6(1):298–306. doi: 10.1089/heq.2021.0076 (PMC9081065; doi:10.1089/heq.2021.0076)

Supplementary Material

Evaluating safety net hospitals: systematic review of operational definitions, recommendations for a new framework, and health policy implications

Supplementary Materials

Table of Contents

**Figure S1. Flow chart of study selection process for systematic review**2

**Figure S2. Frequency distribution of safety net related studies over time through May 16, 2019**3

Figure S1. Flow chart of study selection process for systematic review

Figure S2. Frequency distribution of safety net related studies over time through May 16, 2019


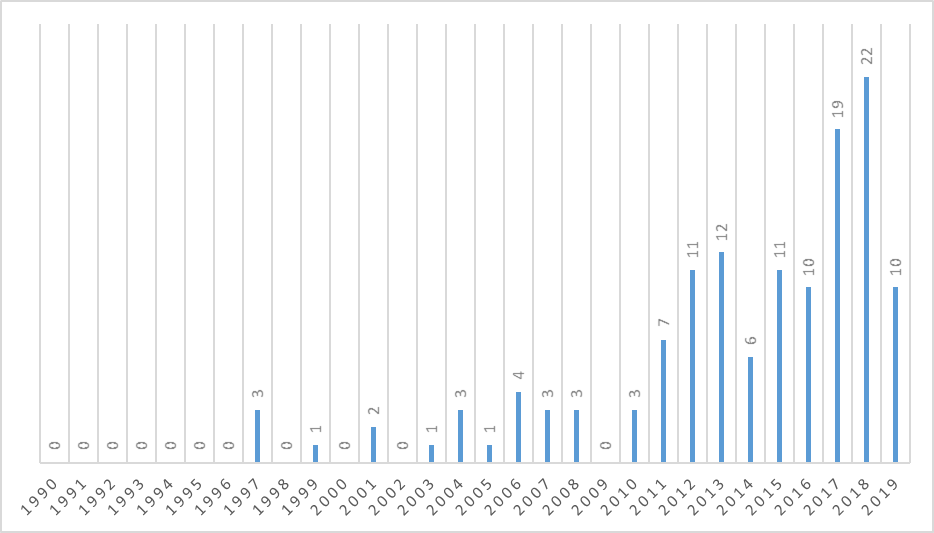

Supplement: Supplemental data [file Suppl_Data.zip › Suppl_Material.docx]
